# Cover cropped and tilled table grape vineyard: Data on leaves and stems

**DOI:** 10.1016/j.dib.2021.106861

**Published:** 2021-02-11

**Authors:** Giuseppe Ferrara, Andrea Mazzeo

**Affiliations:** Department of Soil, Plant and Food Sciences, University of Bari “Aldo Moro”, via G. Amendola 165/A, 70126 - Bari, Italy

**Keywords:** Table grape, Italia, Cover crop, Tillage, Leaves, Shoots, Stem

## Abstract

Data presented are on mass, length, SPAD and some physiological parameters of leaves and stems in a table grape vineyard of Italia variety grafted onto 1103 Paulsen, covered with a plastic sheet to advance ripening and managed with two soil systems in the Puglia region, South-eastern Italy in 2015 and 2016. The two systems differed for the soil management since in one area of the vineyard a cover crop was used (*Trifolium repens* L.), whereas in the other area only soil tillage was adopted. The data of the two seasons include: (a) mass of leaves of primary shoot, secondary shoot and opposite the cluster; (b) length of secondary shoots; (c) number of both secondary shoots and leaves of secondary shoots; (d) SPAD values and area of leaves opposite both first and second cluster on the primary shoot; (e) mass of stems of both primary and secondary shoots; and (f) some physiological parameters (Ψ_stem_, temperature, Fv/Fm). The data in this article support and augment information presented in the research article ‘Cover crops in the inter-row of a table grape vineyard managed with irrigation sensors: effects on yield, quality and glutamine synthetase activity in leaves’ (Sci. Hortic. **281**, 2021 https://doi.org/10.1016/j.scienta.2021.109963).

## Specifications Table

**Table:** 

Subject	Agricultural Sciences
Specific subject area	Viticulture: soil management of the vineyard
Type of data	Table Image Figure Microsoft Excel raw data
How data were acquired	Samples of grapevine shoots were collected in the vineyard at the main physiological stages each season and carried to the lab for the successive analyses. Measurement of fresh mass (f.w.) of leaves and stems was done by using a scale and successively samples of all organs were dried in a ventilated oven (ORMA, model BC, Milan, Italy) at 65 °C until a constant mass for the dry mass determination (d.w.). Leaf area measurements were made with a leaf area meter (LI-3100 area meter, LI-COR Inc., USA). A chlorophyll meter (SPAD-502, Konica Minolta, Japan) was used to make Soil-Plant Analysis Development (SPAD) measurements of leaves (4 per vine) opposite the clusters. Stem water potential (Ψ_stem_) was measured on 3 healthy leaves per vine not exposed to the sun (3 vines per treatment). The leaves were selected and wrapped in polyethylene bags and covered with aluminum foil at least 2 h prior to the measurements. The Ψ_stem_ readings were made at noon with a pump-up chamber (PMS Instrument Company, Albany, OR, USA). Temperatures of both berries and leaves were collected by using a portable infrared thermometer (PCE-777 N, PCE Italia, Capannori, Italy). The Fv/Fm ratio was obtained by using a pocket pea chlorophyll fluorimeter (Hansatech Instruments, Norfolk, UK). Other vegetative data are reported in the related article [1].
Data format	Raw Analyzed
Parameters for data collection	The experimental design adopted in the trial was a single factor (soil management) and two treatments (cover cropped and tilled) of 0.5 ha each. In 2015 and 2016, at each phenological stage according to the BBCH scale (BBCH 65; 71 75; 79; 83; 89) [2], 9 shoots per treatment were sampled and used for the analyses.
Description of data collection	Two soil management systems were evaluated over a two-year period. Grapevine shoots collected at the six main phenological stages in each season were analysed in the lab: length of shoots, leaf area, weight of stems, leaf weight. Measurements of Ψ_stem_, temperatures and fluorescence were performed in the vineyard and data analysed in the lab.
Data source location	Department of Soil, Plant and Food Science (DISSPA), University of Bari ‘Aldo Moro’, Bari, Italy. Location of the table grape vineyard in the countryside of Adelfia (Bari province): lat. 40.970957, long. 16.852581, elevation 218.5 m above sea level.
Data accessibility	Analyzed data: With the article. Raw data: Repository name: MENDELEY DATA. Data identification number: 10.17632/8xzcthp967.1 (Data on leaves and stems of Italia table grape). Direct URL to data: http://dx.doi.org/10.17632/8xzcthp967.1
Related research article	G. Ferrara, D. Nigro, R. Torres, A. Gadaleta, M. W. Fidelibus, A. Mazzeo, Cover crops in the inter-row of a table grape vineyard managed with irrigation sensors: effects on yield, quality and glutamine synthetase activity in leaves, Sci. Hortic. 281, 2021, https://doi.org/10.1016/j.scienta.2021.109963

## Value of the Data

• The data are from a two-year study on the use of a cover crop in the inter-row of a table grape vineyard covered with a plastic sheet to advance ripening. These data give a deep insight on the effects of seeded *Trifolium repens* (white clover) on vegetative parameters such as shoot length, stem weight and leaf area of the table grape variety Italia. Puglia is the most important region in Italy and one of the most important in the world for table grape cultivation.
• The impact of cover crops in table grape vineyards covered with a plastic sheet is still poorly investigated. This data could be valuable for future studies to understand the effects of cover crops on the vegetative responses of both seeded and seedless table grape varieties.
• Data can also be taken into consideration from viticulturists for the use of an inter-row cover crop in similar vineyard conditions to develop a more sustainable management of table grape vineyards.
• The data may serve as a benchmark for future researches aiming at investigating the effects on vine of more sustainable managements of table grape vineyards in areas with a Mediterranean climate.

## 1 Data Description

**Fig. 1:**
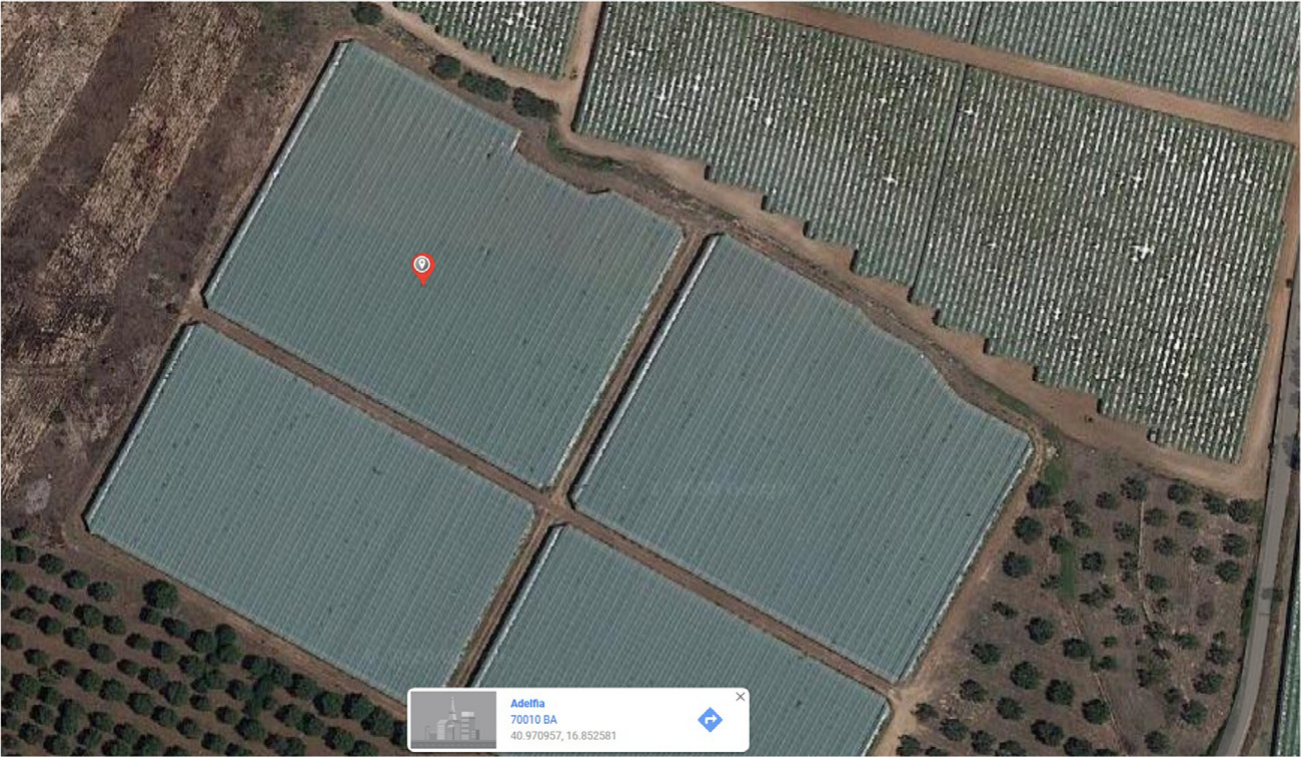
Site localization of the trial with the indication of town, postal code, latitude and longitude (figure was obtained from Google Maps).

**Fig. 2:**
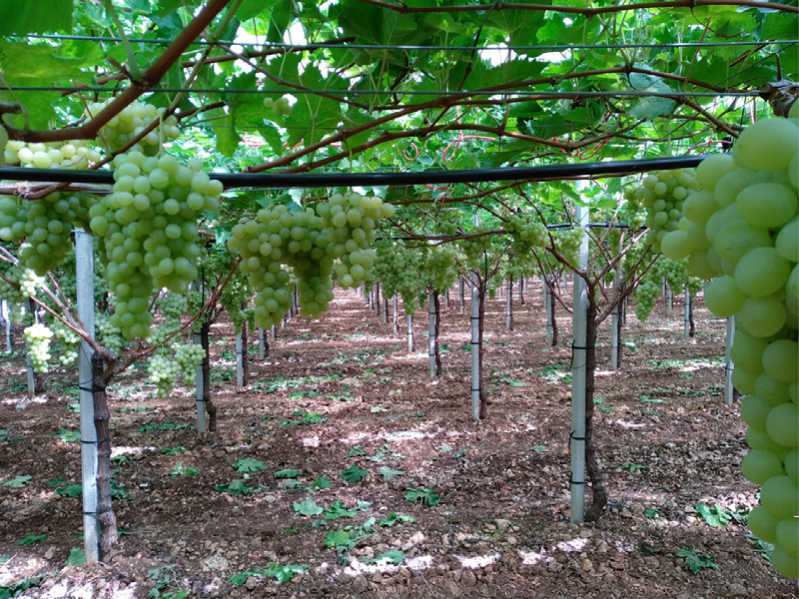
Tilled area of the vineyard.

**Fig. 3:**
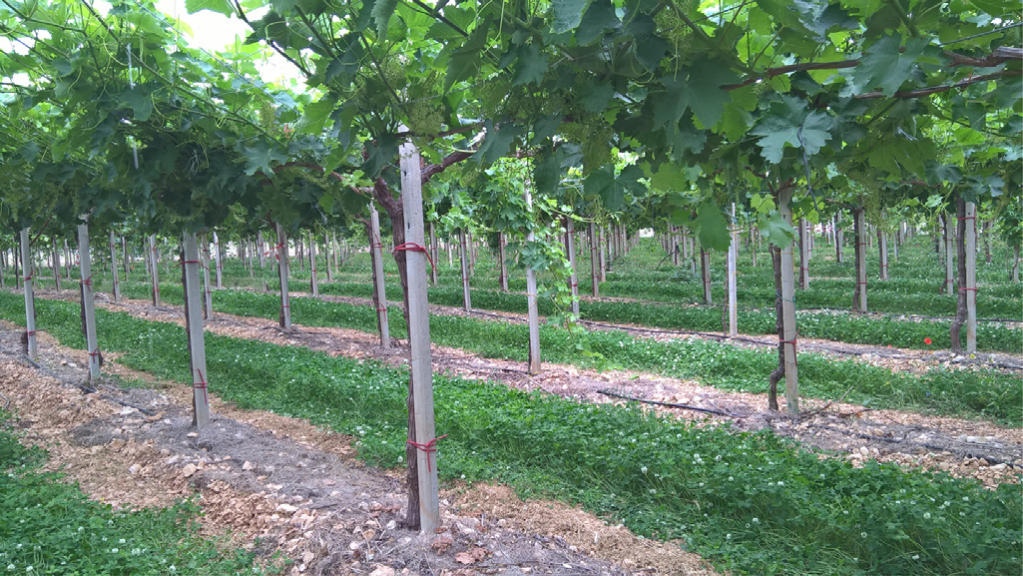
Cover cropped area of the vineyard.

This article includes the raw data, descriptive data (means) and statistics (95% confidence intervals with REGWQ test for stages comparison) on the effects of two soil management systems over a two-year period on the vegetative parameters of Italia table grape variety grown in Puglia region, South-eastern Italy. The data presented here include some vegetative parameters collected in the experiment, whereas the data of other parameters were used in the analyses reported in the related article [1]. Vegetative samples were collected at six phenological stages, according to the BBCH scale [2]. Data include: a) site localization (Fig. 1) and tilled (Fig. 2) and cover cropped (Fig. 3) area of the vineyard at spring-summer; b) mass of the primary leaves in 2015 and 2016 (Table 1); c) mass of leaves opposite the clusters in 2015 and 2016 (Table 2); d) length of the secondary shoots in 2015 and 2016 (Table 3); e) number of both secondary shoots and leaves per secondary shoot in 2015 and 2016 (Table 4); f) mass of the secondary leaves in 2015 and 2016 (Table 5); g) SPAD values and area of leaves opposite both the first and second cluster on the primary shoot (Table 6); h) mass of primary shoot stems in 2015 and 2016 (Table 7); i) mass of secondary shoot stems in 2015 and 2016 (Table 8); l) Ψ_stem_, temperatures of leaves and berries and Fv/Fm (Table 9). The raw data are provided in the Mendeley repository.

**Table 1:** Mass of the primary leaves in 2015 and 2016. Letters within columns indicate significant differences (P < 0.05) between stages and for each parameter according to REGQW test. *, **, *** and ns indicate significance at *P* < 0.05, 0.01, 0.001 and not significant, respectively.

	Leaves/shoot f.w. (g.)				Leaves/vine f.w. (g.)				Leaves/shoot d.w. (g.)				Leaves/vine d.w. (g.)				Water content (%)			
	2015		2016		2015		2016		2015		2016		2015		2016		2015		2016	
Stage	Cover	Tilled	Cover	Tilled	Cover	Tilled	Cover	Tilled	Cover	Tilled	Cover	Tilled	Cover	Tilled	Cover	Tilled	Cover	Tilled	Cover	Tilled
Flowering	84.0 d	81.8 d	62.5 c	66.1 b	2773.2 d	2584.7 d	2107.5 c	2210.0 b	17.2 b	15.7 d	12.7 b	13.6 b	566.8 b	494.8 d	492.2 b	455.3 b	79.6 ab	80.8 a	79.8 a	79.5 a
Berry-set	131.0 cd	148.2 bc	111.0 ab	128.9 a	4324.2 cd	4685.9 bc	3741.2 ab	4310.1 a	23.4 b	28.8 c	24.1 a	27.1 a	773.0 b	909.9 c	811.4 a	905.8 a	82.1 a	80.6 a	77.7 ab	78.9 ab
Berry growth	226.8 a	230.0 a	119.6 ab	159.4 a	7484.1 a	7270.6 a	4032.1 ab	5330.3 a	42.4 a	47.3 a	26.0 a	34.0 a	1400.2 a	1493.6 a	876.0 a	1135.4 a	81.2 a	79.4 ab	78.4 ab	78.6 ab
Veraison	182.1 b	190.3 b	142.7 a	113.1 ab	6010.3 b	6015.0 b	4804.4 a	3783.4 ab	40.1 a	43.6 ab	31.7 a	25.5 a	1323.7 a	1376.8 ab	1067.8 a	851.5 a	77.9 bc	77.1 bc	77.8 ab	77.5 bc
Ripening	138.4 c	147.5 bc	99.7 bc	120.1 ab	4565.9 c	4663.0 bc	3360.7 bc	4017.5 ab	34.4 a	34.3 bc	22.1 ab	28.7 a	1134.9 a	1084.3 bc	743.2 ab	960.6 a	75.1 c	76.7 c	78.2 ab	76.1cd
Harvest	130.4 cd	136.2 c	94.7 bc	114.5 ab	4304.7 cd	4305.4 c	3193.1 bc	3828.1 ab	37.1 a	34.6 bc	22.4 ab	28.8 a	1225.9 a	1094.7 bc	755.9 ab	963.5 a	71.4 d	74.6 c	76.8 b	75.0 d
*Treatment*	*ns*		*ns*		*ns*		*ns*		*ns*		*Ns*		*ns*		*ns*		*ns*		*ns*	
*Year (Cover)*	***				***				***				***				*ns*			
*Year (Tilled)*	***				***				***				***				*ns*

**Table 2:** Mass of leaves opposite the cluster in 2015 and 2016. Letters within columns indicate significant differences (P < 0.05) between stages and for each parameter according to REGQW test. *, **, *** and ns indicate significance at *P* < 0.05, 0.01, 0.001 and not significant, respectively.

	Leaf opposite cluster/shoot f.w. (g.)				Leaf opposite cluster/vine f.w. (g.)				Leaf opposite cluster/shoot d.w. (g.)				Leaf opposite cluster/vine d.w. (g.)				Water content (%)			
	2015		2016		2015		2016		2015		2016		2015		2016		2015		2016	
Stage	Cover	Tilled	Cover	Tilled	Cover	Tilled	Cover	Tilled	Cover	Tilled	Cover	Tilled	Cover	Tilled	Cover	Tilled	Cover	Tilled	Cover	Tilled
Flowering	14.9 b	11.6 b	12.5 b	17.8	491.8 b	367.3 b	422.6 b	594.4	3.0 b	2.3 b	2.7	4.0	100.4 b	73.0 b	90.0	152.6	79.6 ab	80.1 a	78.8	77.7 b
Berry-set	15.2 b	17.6 b	19.6 ab	24.1	502.6 b	555.2 b	661.4 ab	806.5	2.7 b	3.6 ab	4.2	5.1	89.3 b	113.1 ab	141.8	172.1	82.2 a	79.6 a	79.0	78.5 ab
Berry growth	24.1 a	22.8 a	23.7 a	20.5	794.7 a	722.2 a	799.9 a	687.1	4.5 a	4.6 a	4.8	4.2	149.3 a	146.5 a	163.4	141.4	81.2 ab	79.7 a	78.0	80.4 a
Veraison	14.0 b	13.3 b	15.9 ab	19.8	460.4 b	421.8 b	534.7 ab	662.8	3.1 b	3.1 ab	3.5	4.6	102.4 b	98.2 ab	117.7	154.8	77.7 bc	76.7 b	77.9	76.7 bc
Ripening	10.5 b	14.3 b	16.6 ab	16.3	345.9 b	451.9 b	558.3 ab	546.5	2.6 b	3.4 ab	4.0	4.1	86.8 b	108.4 ab	135.3	138.6	75.1 c	76.0 b	76.2	74.1 cd
Harvest	10.0 b	11.2 b	12.8 ab	15.8	330.5 b	353.3 b	432.4 ab	529.1	2.9 b	3.1 ab	3.5	4.5	95.7 b	96.5 ab	117.7	149.1	70.3 d	72.7 b	72.3	71.7 d
*Treatment*	*ns*		*ns*		*ns*		*ns*		*ns*		*Ns*		*ns*		*ns*		*ns*		*ns*	
*Year (Cover)*	*				*				*				*				*ns*			
*Year (Tilled)*	*				**				***				***				*ns*

**Table 3:** Length of the secondary shoots in 2015 and 2016. Letters within columns indicate significant differences (P < 0.05) between stages and for each parameter according to REGQW test. *, **, *** and ns indicate significance at *P* < 0.05, 0.01, 0.001 and not significant, respectively.

	Average length per shoot (cm)				Length of shoots/vine (m)			
	2015		2016		2015		2016	
Stage	Cover	Tilled	Cover	Tilled	Cover	Tilled	Cover	Tilled
Flowering	25.7	24.2 b	31.0	56.2	8.5	7.6 b	10.4	18.8
Berry-set	52.3	72.5 ab	70.9	71.8	17.3	21.9 ab	23.9	57.5
Berry growth	28.7	65.7 ab	59.8	89.4	9.5	20.8 ab	20.1	29.9
Veraison	52.0	74.0 ab	74.8	142.7	17.2	23.4 ab	25.2	47.7
Ripening	51.7	79.4 ab	117.9	120.7	17.1	25.1 ab	39.7	40.4
Harvest	80.3	119.7 a	106.9	97.1	26.5	37.8 a	36.0	32.5
*Treatment*	*		*		*		*	
*Year (Cover)*	*				*			
*Year (Tilled)*	*				**

**Table 4:** Number of both secondary shoots and leaves per secondary shoot in 2015 and 2016. Letters within columns indicate significant differences (P < 0.05) between stages and for each parameter according to REGQW test. *, **, *** and ns indicate significance at *P* < 0.05, 0.01, 0.001 and not significant, respectively.

	Shoots/primary shoot (n.)				Shoots/vine (n.)				Leaves/shoot (n.)				Leaves /vine (n.)			
	2015		2016		2015		2016		2015		2016		2015		2016	
Stage	Cover	Tilled	Cover	Tilled	Cover	Tilled	Cover	Tilled	Cover	Tilled	Cover	Tilled	Cover	Tilled	Cover	Tilled
Flowering	6.7	6.1 b	6.2	6.8	220.0	193.2 b	209.7	226.7	9.2	7.7	7.0	10.8	304.3	242.4	235.9	360.5
Berry-set	9.9	12.0 a	11.7	12.1	325.9	379.3 a	393.2	405.0	17.1	22.7	17.2	31.9	565.1	716.5	580.5	1066.5
Berry growth	7.4	12.3 a	7.8	8.9	245.7	389.9 a	262.1	297.3	11.6	21.1	15.2	20.4	381.3	667.3	513.0	683.8
Veraison	8.6	11.6 ab	8.8	11.3	282.3	367.5 ab	295.8	379.0	16.2	22.8	18.7	27.8	535.3	719.2	629.1	929.0
Ripening	10.7	9.6 ab	10.6	11.1	352.0	302.1 ab	355.8	371.6	15.7	19.8	29.1	27.4	517.0	625.2	981.2	917.9
Harvest	11.0	11.0 ab	8.9	11.4	363.0	347.7 ab	299.1	382.8	23.3	22.2	26.5	28.7	770.0	702.5	893.1	958.7
*Treatment*	*ns*		*ns*		*ns*		*ns*		*ns*		*ns*		*ns*		*ns*	
*Year (Cover)*	*ns*				*ns*				*ns*				*ns*			
*Year (Tilled)*	*ns*				*ns*				*ns*				*

**Table 5:** Mass of the secondary leaves in 2015 and 2016. Letters within columns indicate significant differences (P < 0.05) between stages and for each parameter according to REGQW test. *, **, *** and ns indicate significance at *P* < 0.05, 0.01, 0.001 and not significant, respectively.

	Leaves/shoot f.w. (g.)				Leaves/vine f.w. (g.)				Leaves/shoot d.w. (g.)				Leaves/vine d.w. (g.)				Water content (%)			
	2015		2016		2015		2016		2015		2016		2015		2016		2015		2016	
Stage	Cover	Tilled	Cover	Tilled	Cover	Tilled	Cover	Tilled	Cover	Tilled	Cover	Tilled	Cover	Tilled	Cover	Tilled	Cover	Tilled	Cover	Tilled
Flowering	5.6 b	4.1 b	5.0	10.3	185.0 b	128.6 b	167.9	344.8	1.2 b	0.8 c	1.0 b	2.1	38.0 b	24.9 c	34.4 b	71.4	79.6 ab	80.5 ab	79.5 a	79.8 b
Berry-set	19.7 b	30.3 a	20.8	55.4	650.9 b	962.3 a	699.9	1853.6	3.4 b	5.5 bc	4.5 ab	11.4	110.6 b	174.2 bc	150.7 ab	380.1	82.4 a	81.6 a	78.9 a	79.6 b
Berry growth	17.3 b	36.0 a	28.3	44.3	569.7 b	1137.4 a	953.9	1480.5	3.4 b	7.1 b	6.3 ab	8.3	111.4 b	225.9 b	212.2 ab	277.2	80.4 ab	80.1 ab	77.0 a	83.0 a
Veraison	19.0 b	25.9 a	26.5	58.4	627.9 b	1134.9 a	891.8	1953.8	4.2 b	7.9 b	5.9 ab	13.4	138.7 b	251.3 b	200.4 ab	446.8	78.1 bc	78.1 ab	77.9 a	77.2 bc
Ripening	20.4 b	31.3 a	42.3	48.7	672.0 b	990.8 a	1424.1	1629.9	5.0 b	7.7 b	11.7 a	12.7	165.9 b	244.9 b	394.5 a	426.3	75.4 c	75.3 bc	75.3 ab	74.9 c
Harvest	43.6 a	47.1 a	41.5	38.4	1438.5 a	1489.3 a	1398.2	1285.4	12.9 a	13.6 a	12.0 a	11.9	426.9 a	430.3 a	402.9 a	397.9	69.5 d	72.1 c	72.0 b	69.0 d
*Treatment*	*		*		*		*		*		*Ns*		*ns*		*ns*		*ns*		*ns*	
*Year (Cover)*	*ns*				*ns*				*Ns*				*ns*				*ns*			
*Year (Tilled)*	*ns*				*ns*				*Ns*				*ns*				*ns*

**Table 6:** SPAD values and area of leaves opposite both first and second cluster on the primary shoot. Letters within columns indicate significant differences (P < 0.05) between stages and for each parameter according to REGQW test. *, **, *** and ns indicate significance at *P* < 0.05, 0.01, 0.001 and not significant, respectively.

	SPAD of first leaf				SPAD of second leaf			
	2015		2016		2015		2016	
Stage	Cover	Tilled	Cover	Tilled	Cover	Tilled	Cover	Tilled
Flowering	40.2	39.1 ab	41.2	42.1	39.3 ab	33.1 b	41.3	46.0
Berry-set	42.4	41.5 ab	45.1	44.7	44.0 ab	40.6 a	43.8	44.1
Berry growth	43.5	39.5 ab	45.6	45.4	45.0 a	39.3 a	41.5	40.0
Veraison	39.0	44.0 a	41.7	45.9	41.2 ab	38.6 a	40.2	42.9
Ripening	41.8	38.9 ab	41.9	42.2	42.2 ab	37.4 ab	41.7	42.6
Harvest	43.2	37.1 b	45.1	42.2	37.1 b	41.2 a	41.1	41.9
*Treatment*	*ns*		*ns*		**		*ns*	
*Year (Cover)*	*				*ns*			
*Year (Tilled)*	***				***			

	Leaves opposite the clusters/shoot (cm^2^)				Leaves opposite the clusters/vine (m^2^)			
	2015		2016		2015		2016	
Stage	Cover	Tilled	Cover	Tilled	Cover	Tilled	Cover	Tilled

Flowering	500.8 b	496.6 c	397.1	544.6	1.7 b	1.6 c	1.3	1.8
Berry-set	515.1 b	659.7 b	607.4	682.6	1.7 b	2.1 b	2.0	2.3
Berry growth	654.8 a	803.4 a	660.4	514.3	2.2 a	2.5 a	2.2	1.7
Veraison	627.7 a	619.3 bc	691.2	784.6	2.1 a	2.0 bc	2.3	2.6
Ripening	620.9 a	615.3 bc	557.2	522.2	2.0 a	1.9 bc	1.9	1.7
Harvest	506.5 b	576.9 bc	429.1	547.5	1.7 b	1.8 bc	1.4	1.8
*Treatment*	**		*ns*		*ns*		*ns*	
*Year (Cover)*	*ns*				*ns*			
*Year (Tilled)*	*ns*				*ns*

**Table 7:** Mass of primary shoot stems in 2015 and 2016. Letters within columns indicate significant differences (P < 0.05) between stages and for each parameter according to REGQW test. *, **, *** and ns indicate significance at *P* < 0.05, 0.01, 0.001 and not significant, respectively.

	Average stem f.w. (g.)				Stems/vine f.w. (g.)				Average stem d.w. (g.)				Stems/vine d.w. (g.)				Water content (%)			
	2015		2016		2015		2016		2015		2016		2015		2016		2015		2016	
Stage	Cover	Tilled	Cover	Tilled	Cover	Tilled	Cover	Tilled	Cover	Tilled	Cover	Tilled	Cover	Tilled	Cover	Tilled	Cover	Tilled	Cover	Tilled
Flowering	69.5 b	76.2 b	80.9 b	77.1 b	2292.5 b	2409.9 b	2725.8 b	2577.2 b	11.0 c	10.7 c	12.3 c	11.5 c	362.6 c	339.3 c	413.2 c	383.1 c	84.3 a	86.0 a	84.6 a	85.1 a
Berry-set	123.3 ab	167.0 a	110.7 ab	157.1 ab	4069.5 ab	5279.7 a	3730.7 ab	5253.1 ab	25.1 bc	30.9 bc	19.9 bc	28.3 bc	828.9 bc	975.7 bc	670.5 bc	946.3 bc	80.0 ab	81.7 b	82.0 a	82.0 b
Berry growth	125.5 ab	181.2 a	125.1 ab	156.4 ab	4141.5 ab	5727.4 a	4216.5 ab	5231.3 ab	30.5 b	42.0 b	29.6 bc	36.2 bc	1005.1 b	1328.7 b	997.6 bc	1211.4 bc	76.0 bc	76.7 c	76.9 b	77.1 c
Veraison	137.6 a	172.1 a	157.6 a	202.8 a	4539.7 a	5440.2 a	5310.2 a	6783.8 a	39.5 ab	52.1 ab	40.8 ab	53.2 ab	1304.4 ab	1647.3 ab	1374.9 ab	1779.0 ab	70.1 cd	70.2 d	74.7 b	74.0 c
Ripening	138.2 a	174.8 a	156.0 a	197.0 a	4560.2 a	5526.5 a	5256.2 a	6589.5 a	45.1 ab	60.1 ab	50.9 a	65.9 a	1488.3 ab	1900.0 ab	1714.4 a	2204.3 a	67.6 de	65.9 de	69.2 c	68.0 d
Harvest	136.6 a	186.6 a	152.8 a	188.3 a	4506.8 a	5897.6 a	5148.7 a	6296.5 a	52.8 a	73.4 a	54.3 a	73.0 a	1742.7 a	2319.9 a	1828.5 a	2441.9 a	61.0 e	61.5 e	65.7 d	61.9 e
*Treatment*	***		**		**		*		**		*		*		*		*ns*		*ns*	
*Year (Cover)*	*ns*				*ns*				*ns*				*ns*				*ns*			
*Year (Tilled)*	*ns*				*ns*				*ns*				*ns*				*ns*

**Table 8:** Mass of secondary shoot stems in 2015 and 2016. Letters within columns indicate significant differences (P < 0.05) between stages and for each parameter according to REGQW test. *, **, *** and ns indicate significance at *P* < 0.05, 0.01, 0.001 and not significant, respectively.

	Average stem f.w. (g.)				Stems/vine f.w. (g.)				Average stem d.w. (g.)				Stems/vine d.w. (g.)				Water content (%)			
	2015		2016		2015		2016		2015		2016		2015		2016		2015		2016	
Stage	Cover	Tilled	Cover	Tilled	Cover	Tilled	Cover	Tilled	Cover	Tilled	Cover	Tilled	Cover	Tilled	Cover	Tilled	Cover	Tilled	Cover	Tilled
Flowering	2.3	2.1 b	3.1	6.3	76.8	66.3 b	105.2	210.3	0.3 b	0.3	0.4 b	0.8	10.9 b	9.5	14.3 b	28.1	84.9 a	85.4 a	85.8 a	88.5 a
Berry-set	4.6	7.3 ab	8.1	18.5	150.9	229.9 ab	274.6	618.0	0.7 b	1.1	1.2 ab	2.8	24.6 b	35.0	40.5 ab	92.4	84.1 a	85.0 a	85.3 a	84.7 ab
Berry growth	2.5	7.5 ab	7.9	9.8	81.8	236.9 ab	267.6	328.0	0.5 b	1.3	1.6 ab	1.9	15.4 b	40.1	53.5 ab	63.3	81.5 ab	83.5 a	80.5 b	82.0 bc
Veraison	3.8	7.2 ab	6.6	12.9	125.1	227.7 ab	221.3	429.9	0.8 b	2.4	1.4 ab	2.8	25.2 b	75.0	47.1 ab	92.3	79.0 bc	78.1 b	79.7 b	79.6 bc
Ripening	4.2	7.3 ab	11.2	12.9	138.6	231.9 ab	378.3	432.3	1.1 b	1.7	2.3 ab	3.0	34.8 b	54.2	76.6 ab	101.1	76.8 c	77.2 b	79.4 b	79.7 bc
Harvest	9.9	11.7 a	13.4	10.9	327.4	370.9 a	453.1	364.1	3.3 a	3.5	3.3 a	2.7	109.4 a	111.7	109.6 a	90.0	71.2 d	73.8 b	78.1 b	76.6 c
*Treatment*	*		*ns*		*		*ns*		*ns*		*ns*		*ns*		*ns*		*ns*		*ns*	
*Year (Cover)*	*				*				*ns*				*ns*				**			
*Year (Tilled)*	*				*				*ns*				*ns*				*ns*

**Table 9:** Physiological parameters of Italia table grape in 2015. Letters within columns indicate significant differences (P < 0.05) among seasons according to REGQW test. *, **, *** and ns indicate significance at *P* < 0.05, 0.01, 0.001 and not significant, respectively.

			Temperature leaf		Temperature berry			
	Ψ_stem_ (MPa)		(C°)		(C°)		Fv/Fm	
Stage	Cover	Tilled	Cover	Tilled	Cover	Tilled	Cover	Tilled
Flowering	−0.29 c	−0.19 d	21.2 b	20.8 b	21.3 b	20.4 b	0.81	0.82
Berry-set	−0.38 c	−0.30 c	–	–	–	–	0.83	0.83
Berry growth	−0.36 c	−0.33 c	26.3 a	25.6 a	26.6 a	25.6 a	0.80	0.83
Veraison	−0.52 b	−0.47 b	–	–	–	–	0.83	0.81
Ripening	−0.66 a	−0.58 a	–	–	–	–	0.84	0.84
Harvest	–	–	–	–	–	–	0.83	0.83
*Treatment*	***		*ns*		*		*ns*	

This important two-year study was carried out in the countryside of Adelfia (BA), an area very important and known since more than a century ago for the cultivation of table grape in Italy. The vineyard is only 20 km distant for the Department of Soil, Plant and Food Science (DISSPA), University of Bari ‘Aldo Moro’, Bari, Italy. This ongoing systems study was designed to provide information on the impact of an inter-row cover crop (*Trifolium repens* L.) on the vegetative responses of the table grape variety Italia, the most important seeded variety grown in the region.

## 2 Experimental Design, Materials and Methods

### 2.1 Location and experimental design

The trial was conducted over a two-year period (2015–2016) in two areas of a commercial 10-year old ‘Italia’ table grape variety grafted onto 1103 Paulsen rootstock. The vineyard is located in the countryside of Adelfia (Bari province), Puglia region, South-eastern Italy at GPS coordinates lat. 40.970957, long. 16.852581 and elevation 218.5 m above sea level. Vines spacing 2.2 × 2.8 m and epsilon trellising system with four fruiting canes/vine (40–50 buds/vine). In February, as a traditional practice in the area in order to advance ripening, the vineyard was covered with a plastic sheet 160 µm thick and 150 g/m^2^, with 88% light transmittance and 35% light diffusion. The sheets were removed after harvest and before winter pruning. The data of soil analysis are reported in [1], whereas fertilization schedule and irrigation volumes are reported in [3] and [4], respectively. Apart from soil management and irrigation, all other managements (i.e., pest control, summer pruning, fertilization) were equivalent for the two vineyard areas [1]. Two different vineyard soil management practices were compared, cover crop, as recently introduced in table grape vineyards, and tillage, as the traditional practice adopted in the area. In order to compare the two managements, in the same vineyard (1 ha) two contiguous areas were used. The first trial area was seeded (50 kg/ha) with white clover (*Trifolium repens* L.) only in the inter-row in November 2014, whereas the latter consisted of a traditional tilled soil. The experimental design was a single factor (soil management), two treatments (cover crop and tillage) of around 0.5 ha each. Each treatment consisted of three sampling areas (blocks) of 30 vines each (a total of 90 vines per treatment) with 10 vines per repetition (3 repetitions per block) and 6 tagged vines for the measurements. The tagged vines were characterized by a general uniform crop load and canopy as observed in the summer 2014 and during the growing season. In particular, the 30 vines were arranged in three rows of 10 vines per row (single repetition) separated from each other by at least one row and from the border by at least four rows.

### 2.2 Plant material sampling and analysis

The different annual organs of the vines (leaves and stems of the primary and secondary shoots) were sampled from the middle nodes of selected fruiting canes during the main phenological stages of each season (BBCH 65; 71 75; 79; 83; 89). A total of 9 shoots per treatment were sampled at each phenological stage (3 shoots/sampling area), enclosed in huge plastic bags, and quickly carried to the lab for all the analyses. Before destructive analyses, for each primary shoot, both the secondary shoots and their leaves were counted. The length of the secondary shoots was obtained with the use of a 5 m steel measuring tape self-retract flexible metric tape. Measurement of fresh mass (f.w.) of leaves and stems was done by using a scale. After detaching the leaves, leaf area measurement was made by using a leaf area meter (LI-3100 area meter, LI-COR Inc., USA). Leaf area was expressed as cm^2^ (leaves opposite the clusters), and converted to m^2^ for expressing the area of all the leaves opposite the clusters for each vine (leaf area of the single shoot × all shoots of vine at each stage). Successively samples of leaves and stems were dried in a ventilated oven (ORMA model BC, ORMA s.r.l., Milan, Italy) at 65 °C until a constant mass for the dry mass determination (d.w.). A chlorophyll meter (SPAD-502, Konica Minolta, Japan) was used to make the SPAD measurements of leaves (4 per vine) opposite the clusters. Stem water potential (Ψ_stem_) was measured on 3 healthy leaves per vine not exposed to the sun (9 vines per treatment). The leaves were selected and wrapped in polyethylene bags and covered with aluminum foil at least 2 h prior to the measurements. The Ψ_stem_ readings were made at noon with a pump-up chamber (PMS Instrument Company, Albany, OR, USA). Temperatures of both berries and leaves were collected in the vineyard by using a portable infrared thermometer (PCE-777 N, PCE Italia, Capannori, Italy). The Fv/Fm ratio was obtained in the vineyard by using a pocket pea chlorophyll fluorimeter (Hansatech Instruments, Norfolk, UK).

### 2.3 Statistical analysis

Analysis of variance (ANOVA) was performed with XLSTAT-Pro-software (Addinsoft, Paris, France) at the P < 0.05 for comparison between phenological stages and at P < 0.05, 0.01 and 0.001 for the other analyses (treatment and year). The assumptions of variance were verified with the Levene test (homogeneity of variance) and the Lillefors and Shapiro-Wilk tests (normal distribution). The mean values obtained for the different factors were statistically separated by using the REGWQ test.

## Ethics Statement

The work involved neither the use of human subjects nor animal experiments. Data were not collected from social media platforms.

## CRediT Author Statement

**Giuseppe Ferrara:** Conceptualization, Data curation, Formal analysis, Methodology, Project administration, Resources, Supervision, Visualization, Writing – original draft, Writing –review and editing; **Andrea Mazzeo**: Data curation, Investigation, Methodology, Writing- Original draft preparation, Writing –review and editing.

## Declaration of Competing Interest

The authors declare that they have no known competing financial interests or personal relationships which have or could be perceived to have influenced the work reported in this article.
